# Systematic reviews and meta-analyses for the WHO/ILO Joint Estimates of the Work-related Burden of Disease and Injury

**DOI:** 10.1016/j.envint.2021.106605

**Published:** 2021-10

**Authors:** Frank Pega, Natalie C. Momen, Yuka Ujita, Tim Driscoll, Paul Whaley

**Affiliations:** a Department of Environment, Climate Change and Health, World Health Organization, Geneva, Switzerland; b Labour Administration, Labour Inspection and Occupational Safety and Health Branch, International Labour Organization, Geneva, Switzerland; c Sydney School of Public Health, Faculty of Medicine and Health, University of Sydney, Sydney, NSW, Australia; d Lancaster Environment Centre, Lancaster University, Lancaster, Great Britain and Northern Ireland, United Kingdom; e Editorial Office of Environment International, Great Britain and Northern Ireland, United Kingdom

## 1 The WHO/ILO Joint Estimates of the Work-related Burden of Disease and Injury

Accurate and transparent global health estimates of the work-related burden of disease and injury are key to policy and practice in occupational and workers’ health and safety, at the workplace, enterprise, national, regional and global levels. Governments, workers, employers and other stakeholders require these to design, plan, cost, implement and evaluate effective actions to prevent work-related loss of life and health. Global health estimates from the World Health Organisation (WHO) and the International Labour Organisation (ILO) comply with the strict statistical rules and guidelines of both organizations and are reported according to the *Guidelines for Accurate and Transparent Health Estimates Reporting* (GATHER) (Stevens et al. 2016). Ideally, such estimates should be based on comprehensive and transparent systematic review and synthesis of the latest evidence relating to exposure to occupational risk factors and health outcomes.

This Special Issue presents a series of 15 systematic reviews, developed to support estimation of global exposure to occupational risk factors and the attributable burden of disease. It also presents several novel methodological tools developed specifically for conducting these systematic reviews. The systematic reviews have been produced as a collaborative effort between WHO and ILO, who have been working together since 2016 to produce these estimates. As a collection, they are unique and agenda-setting, presenting a model for a significant step forward in developing occupational and environmental burden of disease estimates. Moreover, they may also provide a suitable evidence synthesis model for producing other global health norms and standards, such as indicators, health risk assessments, and technical guidelines.

In this editorial, we summarise the new processes that have been implemented; identify key innovations in conducting the systematic reviews; highlight their implications for future practice across and beyond the occupational and environmental health and safety domains; and draw out lessons for future estimation of burden of disease and other scenarios where scientific research is reviewed and synthesised to inform policy and practice.

## 2 The general approach

Since 2016, in the spirit of the 2030 Sustainable Development Agenda (United Nations General Assembly 2015), WHO and ILO, the United Nations Specialized Agencies for health and labour, have been working together on the production of the first WHO/ILO Joint Estimates of the Work-related Burden of Disease and Injury (WHO/ILO Joint Estimates). To improve policy coherence and foster partnerships for development, WHO and ILO have combined expertise and resources to produce joint estimation methodologies and one harmonized set of estimates. While established methodologies already existed for estimating the occupational disease burden for 39 established pairs of occupational risk factors and health outcomes, methodologies needed to be developed for 13 selected additional pairs to further improve the coverage of the Global Comparative Risk Assessment (Ezzati et al., 2004, World Health Organization, 2016).

Therefore, to inform generation of estimates for these additional pairs, WHO and ILO produced a series of ten systematic review protocols and conducted 15 systematic reviews and meta-analyses (Table 1). Of the 15 systematic reviews and meta-analyses (Table 1), five were of studies estimating the prevalence and/or level of exposure to occupational risk factors. Ten were of the effect of exposure to occupational risk factors on health outcomes. WHO was an early adopter of systematic reviews and has a long track record of conducting these as the basis for its global health estimates. The systematic reviews for the WHO/ILO Joint Estimates, however, were the first systematic reviews conducted jointly by WHO and ILO on the topic of disease burden.

**Table 1:** WHO/ILO Joint Estimates Systematic Reviews.

	Title	Protocol	Systematic review	Systematic review type
1	The prevalence of occupational exposure to ergonomic risk factors	Hulshof et al. (2019)	Hulshof et al. (2021a)	Prevalence
2	The effect of occupational exposure to ergonomic risk factors on osteoarthritis and other musculoskeletal diseases	Hulshof et al. (2019)	Hulshof et al. (2021b)	Effect
3	The prevalence and level of occupational exposure to dusts and/or fibres	Mandrioli et al. (2018)	Schlünssen et al. (Under review)	Prevalence
4	The effect of occupational exposure to dusts and/or fibres on pneumoconiosis	Mandrioli et al. (2018)	Ongoing	Effect
5	The prevalence of occupational exposure to solar ultraviolet radiation	Paulo et al., 2019, Tenkate et al., 2019	Ongoing	Prevalence
6	The effect of occupational exposure to solar ultraviolet radiation on cataract	Tenkate et al. (2019)	Ongoing	Effect
7	The effect of occupational exposure to solar ultraviolet radiation on melanoma and non-melanoma skin cancer	Paulo et al. (2019)	Ongoing	Effect
8	The prevalence of occupational exposure to noise	Teixeira et al. (2019)	Teixeira et al. (2021a)	Prevalence
9	The effect of occupational exposure to noise on cardiovascular disease	Teixeira et al. (2019)	Teixeira et al. (2021b)	Effect
10	The prevalence of occupational exposure to long working hours	Descatha et al., 2018, Godderis et al., 2018, Li et al., 2018, Rugulies et al., 2019	Ongoing	Prevalence
11	The effect of occupational exposure to long working hours on ischaemic heart disease	Li et al. (2018)	Li et al. (2020a)	Effect
12	The effect of occupational exposure to long working hours on stroke	Descatha et al. (2018)	Descatha et al. (2020)	Effect
13	The effect of occupational exposure to long working hours on depressive disorder	Rugulies et al. (2019)	Rugulies et al. (Under review)	Effect
14	The effect of occupational exposure to long working hours on alcohol use disorders	Godderis et al. (2018)	Pachito et al. (2021)	Effect
15	The effect of occupational exposure to welding fumes on tracheal, bronchus, and lung cancer	Pega et al. (2020a)	Ongoing	Effect

WHO and ILO were supported in the conduct of these systematic reviews by government departments in 11 countries. These included ministries of health and/or labour and national institutes of occupational health and safety, in Brazil, Bulgaria, Denmark, Finland, Italy, Japan, the Netherlands, People’s Republic of China, Republic of Korea, South Africa, and Thailand.

Over 220 individual experts in research organizations, including universities and national academies of science, from 35 countries covering all WHO and ILO regions actively participated. This large collective effort has significantly enhanced capacity in systematic review and burden of disease estimation across policy makers, practitioners and academia, from national to international levels, for better global occupational and workers’ health and safety.

## 3 Innovations

As far as we are aware, this is the most comprehensive use to date of systematic review methods in occupational burden of disease estimation. These are the first applications of a comprehensive systematic review framework in assessing evidence to study the effects of occupational risk factors on health outcomes; it is the first time any occupational or environmental health systematic reviews for such estimation published by WHO or ILO have been conducted according to pre-published, peer-reviewed protocols; and it is the first time that systematic review methods have been applied to assess the prevalence of exposure to occupational risk factors. It is also the first application of systematic review methods within the "Navigation Guide" framework (Woodruff and Sutton, 2014) to synthesising evidence of the effect of a *social* determinant of health, namely exposure to long working hours, on health outcomes.

### 3.1 New tools to support systematic reviews of the prevalence of risk factors

Prior to this work, no framework existed for systematically reviewing studies estimating the prevalence of exposure to risk factors to human health, including occupational or environmental ones. World Health Organization and International Labour Organization (2019b) a Working Group of systematic review methodologists and exposure scientists to support the development of a suite of new tools and approaches for assessing bias and quality of evidence in studies estimating the prevalence of exposure to occupational risk factors, namely protocols for systematic reviews of prevalence studies, the RoB-SPEO tool and the QoE-SPEO approach (Table 2). WHO and ILO, supported by individual experts, developed, pilot-tested, published and applied RoB-SPEO and QoE-SPEO in the five WHO/ILO systematic reviews of prevalence studies (see Table 1). The organizations and their partners are currently evaluating assessor burden, inter-rater reliability and user experience of the RoB-SPEO tool (Momen et al. Submitted). More work is needed to establish a dedicated systematic review framework for prevalence studies of risk factor exposures.

**Table 2:** New methodological tools and approaches for systematic reviews of prevalence studies.

	Methodological tool/approach	Information about the tool/approach	Reference
1	Protocols for the prevalence of exposure to occupational risk factors	Designed to transparently and consistently report planned methods that are standardized across the series of systematic reviews. Pre-publication of a peer-reviewed protocol discourages *ad hoc* decision-making that can bias a review and improves scientific quality by resolving methodological issues prior to conduct of a lengthy, resource-intensive evidence synthesis.	Descatha et al., 2018, Godderis et al., 2018, Hulshof et al., 2019, Li et al., 2018, Mandrioli et al., 2018, Paulo et al., 2019, Rugulies et al., 2019, Teixeira et al., 2019, Tenkate et al., 2019
2	Assessing risk of bias in studies estimating the prevalence of exposure to occupational risk factors (RoB-SPEO)	Designed to aid assessors in judging risk of bias of studies included in a systematic review of prevalence studies. Assessment follows standard practices in healthcare and environmental health systematic review (Higgins and Green, 2011, Rooney et al., 2014, Whaley et al., 2020; Woodruff and Sutton, 2011), facilitating qualitative judgement of the internal validity of studies across eight risk of bias domains.	Pega et al. (2020b)
3	Assessing the quality of evidence in studies estimating prevalence of exposure to occupational risk factors (QoE-SPEO)	Designed to aid assessment of the quality of a body of evidence supporting the findings of a systematic review of prevalence studies. The approach was developed to be consistent with the Grading of Recommendations, Assessment, Development, and Evaluation (GRADE) methodology for assessing certainty of evidence (Guyatt et al 2008; Morgan et al. 2016).	Pega et al. (Under review)

### 3.2 Protocols and two-stage peer-review

Pre-specification of methods in research can reduce the risk of a study being biased by ad-hoc decisions made in knowledge of its likely results. Pre-specification of methods may also discourage publication bias, particularly if there is an in-principle guarantee from a journal to publish the complete study regardless of its results, so long as it reasonably adheres to the planned methods (Chambers and Tzavella 2020). While pre-publication, or at least registration, of protocols is common practice in healthcare systematic reviews, it is less prevalent in the occupational and environmental health sciences. Formal “two-stage peer-review” (Chambers 2019), i.e. peer-review and formal publication (not just registration) of a protocol with in-principle guarantee of publication of the final study is rarer still; as far as we are aware, this is the first time any environmental health journal has adopted this publication model for systematic reviews.

Protocol development has other important benefits. For this project, the provision of opportunities for identifying methodological issues before the systematic reviews had been conducted was particularly valuable. Given that many of the review teams at the outset had limited experience in conducting systematic reviews (necessarily so, as in 2016 when the systematic reviews were commissioned there was limited capacity in the field with these methods), the opportunities for improvement in methods, piloting and training afforded by protocol publication was invaluable for final quality of the reviews.

While pre-specifying and publishing protocols adds time in planning, particularly if teams are inexperienced in systematic review, it greatly reduces risk of critical error — a risk that is correspondingly higher for inexperienced teams. A range of critical issues that would have prohibited publication, such as insufficiently sensitive search strategies, issues with valid application of instruments for assessing risk of bias, and ambiguously formulated objectives, were identified and corrected at protocol stage. This prevented wasted effort on a research product that would not otherwise have been able to support the ultimate objectives of providing an evidence base for official WHO/ILO burden of disease estimates. As experience increases, we expect the time it takes to develop a protocol will reduce. The presence of a high-quality protocol also saves time in the final manuscript write-up as the methods section is already written. We believe our approach reinforces the merit of the two-stage “Registered Reports” model for scientific publishing (Nosek et al., 2018, Nosek and Lakens, 2014) and is a powerful demonstration of the fundamental value of protocols for ensuring the scientific quality of systematic reviews.

### 3.3 Coordinated approach across multiple systematic reviews

WHO and ILO implemented a number of measures to achieve consistency in methodology and quality across the commissioned systematic reviews. These included: basing the reviews on one overarching framework, the “Navigation Guide” (Woodruff and Sutton 2014); providing templates for the protocols and final manuscripts to minimise effort required to achieve consistency in structure and reporting of shared methods across the systematic reviews; development of harmonised data extraction sheets; sharing of peer-review comments between author teams to minimise the expected inconsistencies in coverage of issues across individual rounds of peer-review; conducting training, often with participation of the handling editors from the receiving journal; and organising regular coordination meetings for review leads and members. Before their submission to *Environment International* for publication, the systematic reviews were reviewed by the WHO and ILO technical leads, and after their submission they were handled by a specialist systematic reviews editor — a first for this type of project. The WHO coordinating office and the editorial office held regular project update calls. Where possible, the same peer-reviewers reviewed both the protocol and the relevant final review manuscript. This unusually high level of coordination was a significant factor in the overall coherence and consistency of methods, and is recommended for future projects that involve the conduct of multiple systematic reviews.

### 3.4 Raw data collection

The systematic review teams often needed raw data to recalculate exposure levels according to standards set by WHO and ILO. Our systematic review collection reports in detail the raw data these teams were able to retrieve from original study authors of included studies. This is important as often review authors are unable to gather all the information they require from all included studies in their reviews (Li et al., 2020a, Li et al., 2020b). Only approximately one-third of our requests for such missing data were met. The provision of raw data alongside published manuscripts is essential for the conduct of systematic reviews; our work emphasises the importance of efforts being made in the scientific community to make raw data routinely available (Wilkinson et al. 2016).

## 4 Benefits and lessons learnt

### 4.1 Benefits to burden of disease estimates

This is the first time WHO and ILO have collaborated to produce joint estimates of burden of disease. In the past, the organisations have worked separately to produce different set of estimates. Under this newly established collaboration framework (World Health Organization and International Labour Organization, 2019a), WHO and ILO have been able to engage a large number of governments and individual experts across the regions in the global synthesis and consensus building of evidence for disease burden estimation. Based on the evidence base synthesised in the systematic reviews, the organizations have produced the first WHO/ILO Joint Estimates: estimates of exposure to long working hours and of the attributable burdens of ischemic heart disease and stroke (Pega et al., 2021). This has direct benefits for global policy coherence, with WHO recognising the WHO/ILO Joint Estimates as a Global Public Health Good.

Understanding of the evidence base for occupational burden of disease estimation for 13 new risk factor-outcome pairs has been greatly improved. Systematic review methods have been applied to gather input data for risk factor exposure and disease burden modelling, and risk of bias, quality of evidence and strength of evidence have been assessed. Our new methods for systematic reviews of prevalence studies can also be applied elsewhere, such as in exposure assessment for health risk assessment (International Programme on Chemical Safety and Inter-Organization Programme for the Sound Management of Chemicals 2010). These new tools and approaches have rapidly been adopted and applied in systematic reviews outside of the WHO/ILO Joint Estimates (Al Alawi et al., 2020, Naicker et al., 2021), suggesting they fill a methodological gap and have potential to contribute to improving the quality of systematic reviews (Bero 2020).

Target 8.8 of the Sustainable Development Goals aims to “protect labour rights and promote safe and secure working environments for all workers, including migrant workers, in particular women migrants, and those in precarious employment” (United Nations General Assembly, 2015). The WHO/ILO Joint Estimates could provide an additional indicator to complement the two existing indicators for this target (which cover injuries and level of national compliance with labour rights). To that end, WHO and ILO have proposed to the Inter-Agency and Expert Group that an indicator on the number of deaths from work-related *diseases* produced from the WHO/ILO Joint Estimates could be added to the Sustainable Development Goals indicator system (World Health Organization and International Labour Organization, 2019a).

The training of 220 researchers on systematic review methods by WHO and ILO, greatly increases the global pool of those proficient in up-to-date systematic review methods in occupational health. This should have a general effect on enhancing the quality of conduct and peer-review of systematic reviews and, it is hoped, the reporting of information in primary studies that is needed for the systematic review process. WHO and ILO gathered systematic review methodologists in a global Working Group to strengthen evidence synthesis methods in occupational health. Additionally, national government agencies assisted in this collaboration (such as the National Institute for Occupational Health and Poison Control for the People’s Republic of China and the Ministry of Public Health for Thailand); contributing to the systematic reviews by reviewing evidence from their countries, published in national languages.

### 4.2 Lessons learnt for burden of disease estimates

Reflecting on the experiences of WHO/ILO and the collaborators, we note several lessons learnt for future work on burden of disease estimates. First, burden of disease estimates should be based on systematic reviews and meta-analyses of the relevant bodies of evidence (e.g., on risk factor exposure and on the relative risks of the disease among the exposed compared to the unexposed). Second, risk of bias, quality of evidence and strength of evidence should be assessed in systematic reviews for these estimation studies: our experience demonstrates that they are effective and assist producers of global health estimates in complying with the GATHER guidelines. Third, systematic reviews should be conducted according to pre-specified, peer-reviewed protocols, as a useful step for training, piloting and quality control of methods. Fourth, rigorous but flexible coordination mechanisms should be established under strong technical leadership to facilitate the simultaneous conduct of multiple systematic reviews that engage large, interdisciplinary, complex and international working groups of diverse individual experts. Fifth, standardised text for protocols and reviews should be used, as it leads to consistency and ease of reading; additionally, it aids transparency and ensures that all necessary points are included in each publication to fulfil data needs for estimation. Sixth, project planning should allow a lot of time for the process; the development of the evidence base for the WHO/ILO Joint Estimates comprises complex projects and required training of over 200 people.

## 5 Conclusions

The systematic reviews in the first cycle of the WHO/ILO Joint Estimates have applied a number of novel frameworks, methods, approaches and tools, in both the research and publication processes. These innovations could be adopted during future research projects in the areas of burden of disease, to contribute to accurate and transparent global health estimates. Based on the experience of developing the systematic reviews for the WHO/ILO Joint Estimates, WHO has subsequently commissioned a series of systematic reviews on electromagnetic field exposures using the same model (Verbeek et al. 2021): developing protocols, close specialist systematic review editorial involvement in planning, including a systematic review methods specialist to deliver high quality results, and a coordinated approach across groups for consistency. A new approach for evidence review and synthesis as the basis for producing global health norms and standards has been established that has proven to work.

## Disclaimer

The authors alone are responsible for the views expressed in this article and they do not necessarily represent the views, decisions or policies of the institutions with which they are affiliated.

## Funding

This editorial was prepared with financial support to WHO from: the National Institute for Occupational Safety and Health of the Centres for Disease Control and Prevention of the United States of America (Grant 1E11OH0010676-02; Grant 6NE11OH010461-02–01; and Grant 5NE11OH010461-03–00); the German Federal Ministry of Health (BMG Germany) under the BMG-WHO Collaboration Programme 2020–2023 (WHO specified award ref. 70672); and the Spanish Agency for International Cooperation (AECID) (WHO specified award ref.71208). The funders had no role in decision to publish or preparation of the manuscript.

## Declaration of Competing Interest

The authors declare that they have no known competing financial interests or personal relationships that could have appeared to influence the work reported in this paper.
